# Scraps

**Published:** 1895-09

**Authors:** 


### SCRAPS

PICKED UP BY THE ASSISTANT-EDITOR.

---

**Clinical Records** (12).—Athlete: "Did—I—break it, doctor?" Doctor: "Your arm is crushed, your collar-bone broken, your skull——" Athlete: "But—have—I—broken——" Doctor: "What?" Athlete: "The—record?"

**Medical Missions.**—Interest in medical missions generally must have been quickened by Dr. D. W. Torrance's manly and earnest utterances at the missionary breakfast at the recent British Medical Association meeting. He has part-charge of the work at Tiberias, and many will no doubt be glad to send him some help for it, pecuniary or otherwise. In reference to the work in the Holy Land, Mr. E. W. G. Masterman, the assistant medical officer of the English Mission Hospital in Jerusalem, writes (*Edinb. Med. Miss. Soc. Quart. paper*, Aug., 1895) :—

"Palestine may in a sense be looked upon as the place of origin of all Medical Missions, for there the great Founder of Christianity gave His commission to 'Preach the Gospel and heal the sick.' In modern times, too, 'Medical Missions,' in the modern sense, had almost, if not quite, their first beginning in the establishment of a Medical Mission to Jews in the city of Jerusalem upwards of sixty years ago. At the present time there is no land in the world which has, in proportion to its size, more Medical Missionaries. In spite of this fact, it is the feeling of those most interested in the country that there is room for more, and probably at each Mission station the doctor feels he cannot overtake the work as he should. Apart from the sacred feelings connected with the Holy Land, which make many feel that it is a land which has claims on all Christians as a centre of Missionary effort, there is the outstanding fact that Palestine is one of those lands where Medical Missions hold the key to the hearts of the people. As in all lands under Turkish rule, the preaching of the Gospel is attended with the greatest possible difficulties, and no resources are left untried to prevent the Moslems from hearing the Gospel. This is both from religious and political reasons. Every convert from Mahommedanism is a loss to the Turkish army, for only 'the faithful' can serve as soldiers. Medical Missions are the plan of campaign of this modern crusade, which now attempts, by methods consistent with the Gospel of peace, to accomplish something better than that which was attempted and which failed under the brave but mistaken 'soldiers of the Cross' of crusading times."

**Surgical Instruments of Antiquity.**—Many doctors must have been glad at the Association meeting of the opportunity of renewing their acquaintance with the Oppenheimer collection (see the June, 1895, number of this *Journal*, p. 160), and with Dr. Sambon, who so pleasantly described it there and at Bristol in 1894. I here add some comments on the Naples museum in continuation of those I quoted in the last number from the Rev. Oswald Cockayne's *Leechdoms, Wortcunning, and Starcraft of Early England*. There is further an instrument for tapping the dropsical, described by Celsus (Lib. vii., cap. 15) and Paulus Ægineta (Lib. vi., cap. 50). It was somewhat altered in the middle of the seventeenth century by Petit. An instrument suited to carry off the dropsical humours by a little at a time on successive days, as Celsus (*loc. cit.*) and Paulus Ægineta (*loc. cit.*) recommend, has also been dug up. Rust and hard earth, which cannot safely be removed, have blocked up the canal of the relic and render conclusions less certain. The probe "specillum," μήλη, is reported by Cicero to have been invented by the Arcadian Apollo, who also was the first to bind up a wound (Cicero de Nat. Deor., lib. iii., 22). Seven varieties are figured in the book[1] of Professor Vulpes in one plate, with ends obtuse, spoon-shaped, flat and oval, flat and square, flat and divided. The obtuse knob was πυρήν; the spoon was κυάθισκος; those which had a flat extremity were σπαθόμηλαι; such as had a knob at each end were διπύρηνα. The catheter of the ancients is figured by the same writer. It was furnished with a bit of wood to be drawn out by a thread (Galen, Medicus, cap. xix.), to prevent the obstructive effects of capillary attraction and to fetch the urine after it when withdrawn. It is of bronze, and elastic catheters seem to be of modern invention. They have, or had in 1847, eighty-nine specimens of pincers in the Naples museum; fifteen are like what are now called anatomical pincers, one only has the form of the tenaculum, seventeen are depilatory pincers. One pair of nippers is rectilinear, terminating in points like a pair of compasses. Their names were λαβίδες, volsellæ. Hooks, hamuli, ἄγχιστρα, to the number of fourteen, had been laid up in the cases in 1847; also a trident for cauterising (Paulus Ægineta, lib. vi., cap. 48), and a spatula. A silver lancet was accompanied in the excavating by a small spoon, suited, as medical men agree, for examining a small quantity of the flowing blood. There are also cupping vessels of a somewhat spherical shape, from

[1] For review of this, see *Dublin Quart. J. M. Sc.*, vol. xiv., 1852, p. 130.

which air was exhausted by burning a little tow; a flem for bleeding horses, of the same shape as that now used; and a bent lever of steel, μοχλικόν, vectiarius, for raising the bones of the cranium in case of depression by fracture. Professor Vulpes has given us figures of eight steel or iron knives for various surgical purposes, and of a small plate, suitable in the form of its handle for the application of cautery by fire.

**Medical Philology** (XV.)—The *Promptorium* (1440) has "Chavylbone, or chawlbone. *Mandibula*," and also "Iowe, or chekebone. *Mandibula*." It carefully distinguished between Iowe and "Iol, or heed. *Caput*," although there is plenty of evidence that at times jowl and jaw were used interchangeably. Pynson's printed edition in 1499 read "chaule bone," and a 1498 MS. has "iovwe." The *Catholicon* (1483) has "a Chafte; *maxilla, mala, faux, mandubila, mandula, mola; maxillaris, participium*," and under "a Chawyll*e* (Chavyll*e*") and "a Chekebone" refers to this definition. Mr. Way in the Camden Society edition of the *Promptorium*, and Mr. Herrtage in his edition of the *Catholicon*, both have notes on these words. The examples I will quote, re-arranging them as nearly as I can in chronological order. "In the Cursor Mundi [c. 1280] David, when stating how he had killed a lion and a bear, says (ll. 7505–10)

'I had na help bot me allan . . . And scok þam be þe berdes sua

And I laid hand on þaim beleue þat I þair *chaftes* raue in tua,'

where the Fairfax MS. reads *chauelis*, and the Göttingen and Trinity MSS. *chaulis*. Halliwell quotes from MS. Cott. Vespas. A. iii. leaf 7:

'With the *chafte-ban* of a ded has Men sais that therwit slan he was.'

[This, a version of lines 1073-4 of the Cursor Mundi, alludes to the murder of Abel.] In Wright's Political Poems [this should be Songs, not Poems] (Camden Soc.), p. 240, we find 'to *chawle* ne to chyde' [temp. Ed. II.], *i.e.* to jaw, find fault. In the Anturs of Arthur [c. 1370] ([Three Metrical Romances], Camden Soc., ed. Robson), xi. 2, we read:

'Alle the herdus myȝtun here, the hyndest of alle,
Off the *schaft* and the shol, shaturt to the skin.'

[The last word should be shin = chin.] In Sloane MS. [? 15th cent.] 1571, leaf 48b, is given a curious prescription 'for bolnynge vndur þe *chole*,' the principal ingredient of which is a fat cat. In Ywaine & Gawin [c. 1400], 1991, is:

'He strake the dragon in at the *chavyl*, That it came out at the navyl.'

See also E. E. Alliterative Poems, ed. Morris, p. 100, l. 268:

'With this chavyl-bon I xal slo the.' Cov. Myst. Cain & Abel [1468], p. 37.

Gawin Douglas, describing the Trojans on their first landing in Italy, tells how they

'With thare handis brek and *chaftis* gnaw The crustis, and the coffingis all on raw.'
*Eneados* [1513], Bk. vii., l. 250.

'*Brancus*. A gole or a chawle.' Vocabulary, MS. Harl. 1002. In the Master of Game, MS. Vespas. B. xii., leaf 34b, mention is made of the '*iawle-bone*' of a wild boar. '*Bucca, mala inferior*. The cheeke, iawe or iowll.' Junius. 'Chawe bone, *machovere*,' Palsgrave [1530.] Coverdale's Bible, 1535, reads, 'smyte the chaft bones of the lyons whelpes,' *Ps.* lvii. (A.V. lviii.), 6. 'I will open my mouth, and my tonge shal speake out of my chawes,' *Job* xxxiii. 2. In 1597 Peter Lowe, in his *Chyrurgerie*, has 'underneath the chaft bone.' Chaw-bone is met with almost as late as 1670. 'Chaftis, Chafts, the chops. Chaft-blade, the jaw-bone. Chaft-tooth, a jaw-tooth.' Jamieson, [1808.] A. S. *ceafl*. S. Saxon, *cheuele*."

The connection between chavyl and jaw—as shown in the plausible series of chavyl, chawle, chawe, jaw—is one that is not allowed by modern authorities. Prof. Skeat, in the second edition of his *Etymological Dictionary*, says: "I now believe that the words *jowl* and *chaps*, though allied to each other, are entirely unconnected with *jaw*." The *New English Dictionary* says that chavel is "a typical ME. form of the word now written Jowl, jaw-blade, cheek." It cites the Swedish *käft* (pronounced *chäft*), and the Danish *kieft*, chops, and says that chaft is possibly from a stem *kef-*, *kaf-*, "to make a chewing movement with the under jaw."

I would not dare to call in question the statements of these authorities, but would venture to suggest that sufficient importance has not been attached to the differentiation in the *Promptorium* of "Iol" and "Iowe."

